# Supplementary material for: Acceptability and feasibility of long-term, real-time electronic adherence monitoring of HIV pre-exposure prophylaxis (PrEP) use among young women in Kenya: A mixed methods study
Source: PLoS One. 2024 Mar 7;19(3):e0299168. doi: 10.1371/journal.pone.0299168 (PMC10919630; doi:10.1371/journal.pone.0299168)
Supplement: Attachment — Submitted filename: Response to Reviewers.docx [file pone.0299168.s002.docx]

**KENYA MEDICAL RESEARCH INSTITUTE**

Centre for Microbiology Research, P.O. Box 19464 - 00202, NAIROBI - Kenya,

Tel: (254) (020) 2720794, 2720038, Nairobi E-mail: cmr@kemri-nuitm.or.ke Website: www.kemri.org

11^th^ December 2023

To Jill Blumenthal
Academic Editor
PLOS ONE

Carlyle House, Carlyle Road

Cambridge, CB4 3DN

United Kingdom

Dear Dr. Jill Blumenthal,

**RE:** **Response to Review for Manuscript PONE-D-22-24605**

Thank you for your review of our manuscript entitled **“Acceptability and feasibility of long-term, real-time electronic adherence monitoring of HIV pre-exposure prophylaxis (PrEP) use among young women in Kenya: A mixed methods study”** and the opportunity to resubmit a revised version.

We appreciate your and the reviewers’ valuable feedback, and we are pleased to incorporate these recommendations. We are resubmitting the revised manuscript that addresses all concerns. Below is an updated point-by-point response to the comments along with a description of the changes that have been made with reference to the tracked document. The reviewer comments are included in bold text, and our responses are in standard font. We appreciate, in advance, your time and consideration of this revised manuscript for publication in PLOS One.

Thank you very much for the opportunity to address these suggestions and concerns and to revise our manuscript. We look forward to a favorable consideration.

Sincerely,

Bernard Rono on behalf of all the authors

Centre for Microbiology research,

Kenya Medical Research Institute, Nairobi, Kenya

Email:bkrono@kemri-rctp.org

Tel: +254 725026553

**Editor’s comments**

1. **Please ensure that your manuscript meets PLOS ONE's style requirements, including those for file naming.**

We thank the Editor for the important guidance. We have ensured that the manuscript meets PLOS ONE's style requirements.

2. **Please include a complete in your revised copy of PLOS’ questionnaire on inclusivity in global research manuscript.**

We have included copy of PLOS’ questionnaire on inclusivity in global research.

3. **Thank you for stating the following in the Competing Interests section:**

**"I have read the journal's policy and the authors of this manuscript have the following competing; JMB is currently an employee of Gilead Sciences, outside of the present work. JEH reports consultation fees from Merck and stock ownership in Natera. KN has received research funds from Merck (MSD) and speaker fees from Gilead. EAB reports scientific advisory engagement fees and research support from Merck (MSD). The other authors declare no conflicts of interest." Please confirm that this does not alter your adherence to all PLOS ONE policies on sharing data and materials, by including the following statement: "This does not alter our adherence to PLOS ONE policies on sharing data and materials.”**

We have confirmed that the competing interest does not alter our adherence to all PLOS ONE policies on sharing data and materials and have included the following statement: "This does not alter our adherence to PLOS ONE policy on sharing data and materials.”

4. **In your Data Availability statement, you have not specified where the minimal data set underlying the results described in your manuscript can be found.**

"We have updated the Data Availability statement as follows, but would be happy to see your version if preferable: “The study protocol (including analysis plan), data dictionary, and individual participant data that underlie the quantitative results reported in this Article have been de-identified and posted on the Harvard Dataverse. Because qualitative data cannot be fully de-identified, only the qualitative codebook has been posted on the Harvard Dataverse. Transcript data may be shared through appropriate data use agreements by contacting the Senior Program Manager for Research at the Massachusetts General Hospital Center for Global Health ([legarrison@mgh.harvard.edu](mailto:legarrison@mgh.harvard.edu)).”

5. **Your ethics statement should only appear in the Methods section of your manuscript. If your ethics statement is written in any section besides the Methods, please move it to the Methods section and delete it from any other section. Please ensure that your ethics statement is included in your manuscript, as the ethics statement entered into the online submission form will not be published alongside your manuscript.**

We thank the Editor for this point. We have moved the ethics statement to the method section of the paper.

***Reviewer #1****:*

Dear Author,

Thank you for this study

It is a very good addition to the previous studies/ research on prevention of HIV.
This is a highly studious work with excellent originality.

Abstract: It is interesting and validly comprises of the right expectation in the body of the study. Thank you.

Introduction: The social and scientific values of the study was apparent, and the expected bridging function was clear

Methodology: The study design, setting and procedure follow all the necessary rules for the mixed study.

Analysis: Analysis was comprehensive and reasonable. No conflict noticed. The rules guiding the UTATUT in scientific studies were delicately respected.

Discussion: The discussions were original and support with the findings in the study

I concluded that this is a well conducted study, very relevant and specific.
 **1. Please find attached: most grammatical errors.**

We thank the Reviewer for the positive observations about our study. We have taken note and corrected all grammatical errors in the revised version of the paper*.*

**Reviewer #2:**

This is a well-written and important paper that adds to the literature around adherence monitoring for PrEP by assessing young people aged 18-24 at high risk of HIV in Kenya. The analysis and results were well laid-out, and use of the Unified Theory Acceptance and Use of Technology (UTAUT) model provided a clear structure for reporting of qualitative results (Fig 1). The quotes are informative and lend valuable insight to the quantitative data. I have some minor suggestions/comments below:

1. **Abstract line 43 is confusing as written "high levels of usefulness interest"; please clarify**

We have clarified the sentence to read, “Most participants reported high levels of usefulness and high interest in using the monitor with few problems or worries reported throughout follow-up.”

2. **Consider merging Tables 2 and 3 (both Acceptability results) so that the data in Table 2 appears as a 4th column in (current) Table 3 (all sites)**

Table 2 and Table 3 have been merged as recommended and labelled Table 2.

3. **You mention in Study Procedures pg 6 that participants in the Intervention arm had the option to switch from daily SMS reminders to reminders triggered by lack of monitor opening. Can you include in Results the proportion of participants who opted to do this? It's an interesting and valuable data point in itself.**

We appreciate this suggestion and have added the following statement on line 242, “A total of 24 participants switched from daily SMS reminders to those triggered by lack of monitor openings.”

4. **Please include a discussion of whether** **participants could open the bottle but not take the pill, and whether this was assessed as a potential limitation to use of Wisepill devices to accurately measure adherence to PrEP. If this was not addressed at all in this study, please state this as well.**

We have revised a statement in discussion, line 530, to read, “The primary limitation of the study is the potential for social desirability bias; the participants’ knowledge that they were being monitored could have triggered device openings that did not correspond to true PrEP adherence (i.e., pill ingestion).”
